# Supplementary material for: Different functional connectivity gradients reflect aging and Alzheimer’s disease
Source: Nat Neurosci. 2026 Oct 1;29(11):2835–47. doi: 10.1038/s41593-026-02402-0 (PMC13724280; doi:10.1038/s41593-026-02402-0)
Supplement: Supplementary file 1 [file 41593_2026_2402_MOESM1_ESM.pdf]

nature neuroscience

Supplementary information

https://doi.org/10.1038/s41593-026-02402-0

Different functional connectivity gradients reflect aging and Alzheimer's disease

In the format provided by the authors and unedited

Supplementary Material

Model	Cohort	Filter	Method	Thresh	Term	SA axis	RE axis
FC ~ age + pathology	BioFINDER	Full sample	PCA	0.00	Age	$r = 0.22$, $p_{spin} = 0.080$	$r = 0.75$, $p_{spin} < 0.001$
					Pathology	$r = 0.74$, $p_{spin} < 0.001$	$r = 0.24$, $p_{spin} = 0.014$
				0.25	Age	$r = 0.2$, $p_{spin} = 0.083$	$r = 0.77$, $p_{spin} < 0.001$
					Pathology	$r = 0.76$, $p_{spin} < 0.001$	$r = 0.13$, $p_{spin} = 0.176$
				0.50	Age	$r = 0.23$, $p_{spin} = 0.071$	$r = 0.61$, $p_{spin} < 0.001$
					Pathology	$r = 0.75$, $p_{spin} < 0.001$	$r = 0.25$, $p_{spin} = 0.003$
				0.75	Age	$r = 0.46$, $p_{spin} = 0.004$	$r = 0.45$, $p_{spin} < 0.001$
					Pathology	$r = 0.54$, $p_{spin} < 0.001$	$r = 0.31$, $p_{spin} = 0.002$
			DME	0.00	Age	$r = 0.18$, $p_{spin} = 0.124$	$r = 0.8$, $p_{spin} < 0.001$
					Pathology	$r = 0.67$, $p_{spin} < 0.001$	$r = 0.25$, $p_{spin} = 0.016$
				0.25	Age	$r = 0.19$, $p_{spin} = 0.089$	$r = 0.8$, $p_{spin} < 0.001$
					Pathology	$r = 0.73$, $p_{spin} < 0.001$	$r = 0.2$, $p_{spin} = 0.072$
				0.50	Age	$r = 0.21$, $p_{spin} = 0.070$	$r = 0.69$, $p_{spin} < 0.001$
					Pathology	$r = 0.76$, $p_{spin} < 0.001$	$r = 0.37$, $p_{spin} < 0.001$
				0.75	Age	$r = 0.31$, $p_{spin} = 0.020$	$r = 0.52$, $p_{spin} < 0.001$
					Pathology	$r = 0.71$, $p_{spin} < 0.001$	$r = 0.38$, $p_{spin} < 0.001$
	ADNI		PCA	0.00	Age	$r = 0.09$, $p_{spin} = 0.336$	$r = 0.68$, $p_{spin} < 0.001$
					Pathology	$r = 0.54$, $p_{spin} < 0.001$	$r = 0.31$, $p_{spin} = 0.001$
				0.25	Age	$r = 0.09$, $p_{spin} = 0.342$	$r = 0.67$, $p_{spin} < 0.001$
					Pathology	$r = 0.54$, $p_{spin} < 0.001$	$r = 0.3$, $p_{spin} = 0.002$
				0.50	Age	$r = 0.1$, $p_{spin} = 0.315$	$r = 0.65$, $p_{spin} < 0.001$
					Pathology	$r = 0.56$, $p_{spin} < 0.001$	$r = 0.28$, $p_{spin} = 0.002$
				0.75	Age	$r = 0.14$, $p_{spin} = 0.328$	$r = 0.68$, $p_{spin} < 0.001$
					Pathology	$r = 0.62$, $p_{spin} < 0.001$	$r = 0.29$, $p_{spin} < 0.001$
			DME	0.00	Age	$r = 0.14$, $p_{spin} = 0.146$	$r = 0.64$, $p_{spin} < 0.001$
					Pathology	$r = 0.52$, $p_{spin} < 0.001$	$r = 0.3$, $p_{spin} = 0.001$
				0.25	Age	$r = 0.13$, $p_{spin} = 0.154$	$r = 0.64$, $p_{spin} < 0.001$
					Pathology	$r = 0.53$, $p_{spin} < 0.001$	$r = 0.3$, $p_{spin} = 0.001$
				0.50	Age	$r = 0.14$, $p_{spin} = 0.181$	$r = 0.62$, $p_{spin} < 0.001$
					Pathology	$r = 0.54$, $p_{spin} < 0.001$	$r = 0.26$, $p_{spin} = 0.001$
				0.75	Age	$r = 0.16$, $p_{spin} = 0.256$	$r = 0.63$, $p_{spin} < 0.001$
					Pathology	$r = 0.59$, $p_{spin} < 0.001$	$r = 0.24$, $p_{spin} = 0.005$

FC ~ age*(-)mPACC + pathology	BioFINDER	cognitively healthy	PCA	0.00	Age	r = 0.22, p_{spin} = 0.098	r = 0.62, p_{spin} < 0.001
					-mPACC	r = 0.07, p_{spin} = 0.196	r = 0.47, p_{spin} < 0.001
					Pathology	r = 0.78, p_{spin} < 0.001	r = -0.04, p_{spin} = 0.419
					Age × -mPACC	r = 0.65, p_{spin} < 0.001	r = -0.03, p_{spin} = 0.265
				0.25	Age	r = 0.21, p_{spin} = 0.094	r = 0.66, p_{spin} < 0.001
					-mPACC	r = 0.09, p_{spin} = 0.158	r = 0.37, p_{spin} < 0.001
					Pathology	r = 0.8, p_{spin} < 0.001	r = -0.11, p_{spin} = 0.249
					Age × -mPACC	r = 0.64, p_{spin} < 0.001	r = 0.03, p_{spin} = 0.543
				0.50	Age	r = 0.21, p_{spin} = 0.115	r = 0.5, p_{spin} < 0.001
					-mPACC	r = 0.1, p_{spin} = 0.130	r = 0.54, p_{spin} < 0.001
					Pathology	r = 0.78, p_{spin} < 0.001	r = -0.02, p_{spin} = 0.458
					Age × -mPACC	r = 0.61, p_{spin} < 0.001	r = -0.1, p_{spin} = 0.098
				0.75	Age	r = 0.41, p_{spin} = 0.034	r = 0.34, p_{spin} = 0.002
					-mPACC	r = 0.02, p_{spin} = 0.444	r = 0.6, p_{spin} < 0.001
					Pathology	r = 0.56, p_{spin} < 0.001	r = 0, p_{spin} = 0.516
					Age × -mPACC	r = 0.52, p_{spin} < 0.001	r = -0.11, p_{spin} = 0.088
			DME	0.00	Age	r = 0.2, p_{spin} = 0.114	r = 0.68, p_{spin} < 0.001
					-mPACC	r = -0.01, p_{spin} = 0.510	r = 0.38, p_{spin} < 0.001
					Pathology	r = 0.71, p_{spin} < 0.001	r = -0.05, p_{spin} = 0.396
					Age × -mPACC	r = 0.68, p_{spin} < 0.001	r = 0.04, p_{spin} = 0.474
				0.25	Age	r = 0.21, p_{spin} = 0.094	r = 0.69, p_{spin} < 0.001
					-mPACC	r = 0.03, p_{spin} = 0.314	r = 0.34, p_{spin} < 0.001
					Pathology	r = 0.76, p_{spin} < 0.001	r = -0.09, p_{spin} = 0.304
					Age × -mPACC	r = 0.68, p_{spin} < 0.001	r = 0.09, p_{spin} = 0.252
				0.50	Age	r = 0.22, p_{spin} = 0.078	r = 0.58, p_{spin} < 0.001
					-mPACC	r = 0.07, p_{spin} = 0.207	r = 0.5, p_{spin} < 0.001
					Pathology	r = 0.77, p_{spin} < 0.001	r = 0.01, p_{spin} = 0.414
					Age × -mPACC	r = 0.68, p_{spin} < 0.001	r = 0, p_{spin} = 0.560
				0.75	Age	r = 0.3, p_{spin} = 0.041	r = 0.43, p_{spin} < 0.001
					-mPACC	r = 0.06, p_{spin} = 0.248	r = 0.58, p_{spin} < 0.001
					Pathology	r = 0.72, p_{spin} < 0.001	r = 0.02, p_{spin} = 0.416
					Age × -mPACC	r = 0.67, p_{spin} < 0.001	r = -0.04, p_{spin} = 0.318

FC ~ age + pathology + -mPACC	impaired (MCI and AD)	PCA	0.00	Age	$r = -0.27$, $p_{spin} = 0.004$	$r = 0.43$, $p_{spin} < 0.001$
				Pathology	$r = -0.28$, $p_{spin} = 0.038$	$r = -0.02$, $p_{spin} = 0.510$
				-mPACC	$r = 0.67$, $p_{spin} < 0.001$	$r = 0.21$, $p_{spin} = 0.042$
			0.25	Age	$r = -0.3$, $p_{spin} = 0.002$	$r = 0.49$, $p_{spin} < 0.001$
				Pathology	$r = -0.25$, $p_{spin} = 0.064$	$r = -0.18$, $p_{spin} = 0.126$
				-mPACC	$r = 0.67$, $p_{spin} < 0.001$	$r = 0.23$, $p_{spin} = 0.064$
			0.50	Age	$r = -0.23$, $p_{spin} = 0.025$	$r = 0.3$, $p_{spin} = 0.002$
				Pathology	$r = -0.22$, $p_{spin} = 0.108$	$r = 0.11$, $p_{spin} = 0.088$
				-mPACC	$r = 0.66$, $p_{spin} < 0.001$	$r = 0.15$, $p_{spin} = 0.062$
			0.75	Age	$r = 0.07$, $p_{spin} = 0.383$	$r = 0.2$, $p_{spin} = 0.081$
				Pathology	$r = -0.4$, $p_{spin} = 0.002$	$r = 0.25$, $p_{spin} = 0.002$
				-mPACC	$r = 0.59$, $p_{spin} < 0.001$	$r = 0.08$, $p_{spin} = 0.136$
		DME	0.00	Age	$r = -0.25$, $p_{spin} = 0.008$	$r = 0.54$, $p_{spin} < 0.001$
				Pathology	$r = -0.33$, $p_{spin} = 0.003$	$r = -0.07$, $p_{spin} = 0.300$
				-mPACC	$r = 0.64$, $p_{spin} < 0.001$	$r = 0.24$, $p_{spin} = 0.031$
			0.25	Age	$r = -0.27$, $p_{spin} = 0.004$	$r = 0.53$, $p_{spin} < 0.001$
				Pathology	$r = -0.28$, $p_{spin} = 0.030$	$r = -0.13$, $p_{spin} = 0.195$
				-mPACC	$r = 0.66$, $p_{spin} < 0.001$	$r = 0.26$, $p_{spin} = 0.031$
			0.50	Age	$r = -0.27$, $p_{spin} = 0.004$	$r = 0.39$, $p_{spin} < 0.001$
				Pathology	$r = -0.25$, $p_{spin} = 0.061$	$r = 0.14$, $p_{spin} = 0.064$
				-mPACC	$r = 0.66$, $p_{spin} < 0.001$	$r = 0.22$, $p_{spin} = 0.009$
			0.75	Age	$r = -0.16$, $p_{spin} = 0.058$	$r = 0.25$, $p_{spin} = 0.028$
				Pathology	$r = -0.33$, $p_{spin} = 0.010$	$r = 0.23$, $p_{spin} = 0.006$
				-mPACC	$r = 0.66$, $p_{spin} < 0.001$	$r = 0.17$, $p_{spin} = 0.029$

Supplementary Table 1: Gradient derivation method and FC thresholding has limited effect on the main results. This table shows the main analyses from Figure 2 and Figure 5 using gradients derived with principal component analysis (PCA) or diffusion map embedding (DME) over four different thresholding values: 0.00, 0.25, 0.50 and 0.75. A threshold of 0.75 means that 75% of the lowest values in the connectivity matrix is zeroed out. In the columns SA axis and RE axis, the Pearson correlation (r) is shown with accompanying p-value from spin tests (one-sided; see Methods), quantifying the relationship between the t-values for each specific term and the gradient scores based on the derivation method and thresholding specified in those two columns. Gradients were derived in all analyses from the average connectome of individuals without cognitive impairment, apoe4 non-carriers without abnormal amyloid levels. In BioFINDER only individuals ≤60 years of age were included while in ADNI the whole age range was used due to the smaller sample size. For the DME method this matrix is then converted into an affinity matrix by taking the cosine similarity between each pair of connectivity profiles, the components are then calculated as outlined in[1].

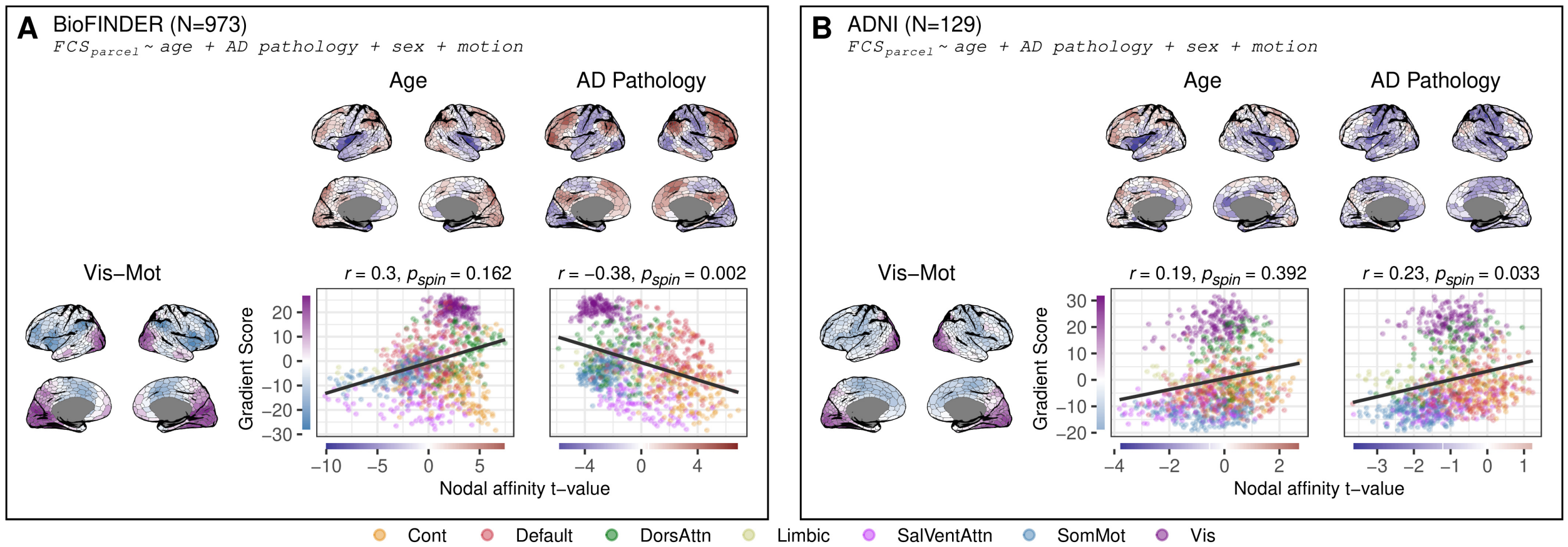

Supplementary Fig. 1: Main results of Figure 2 shown with Gradient 2. This analysis showed that there was no strong or reproducible relationships between the the t-maps for age and AD pathology and Gradient 2.

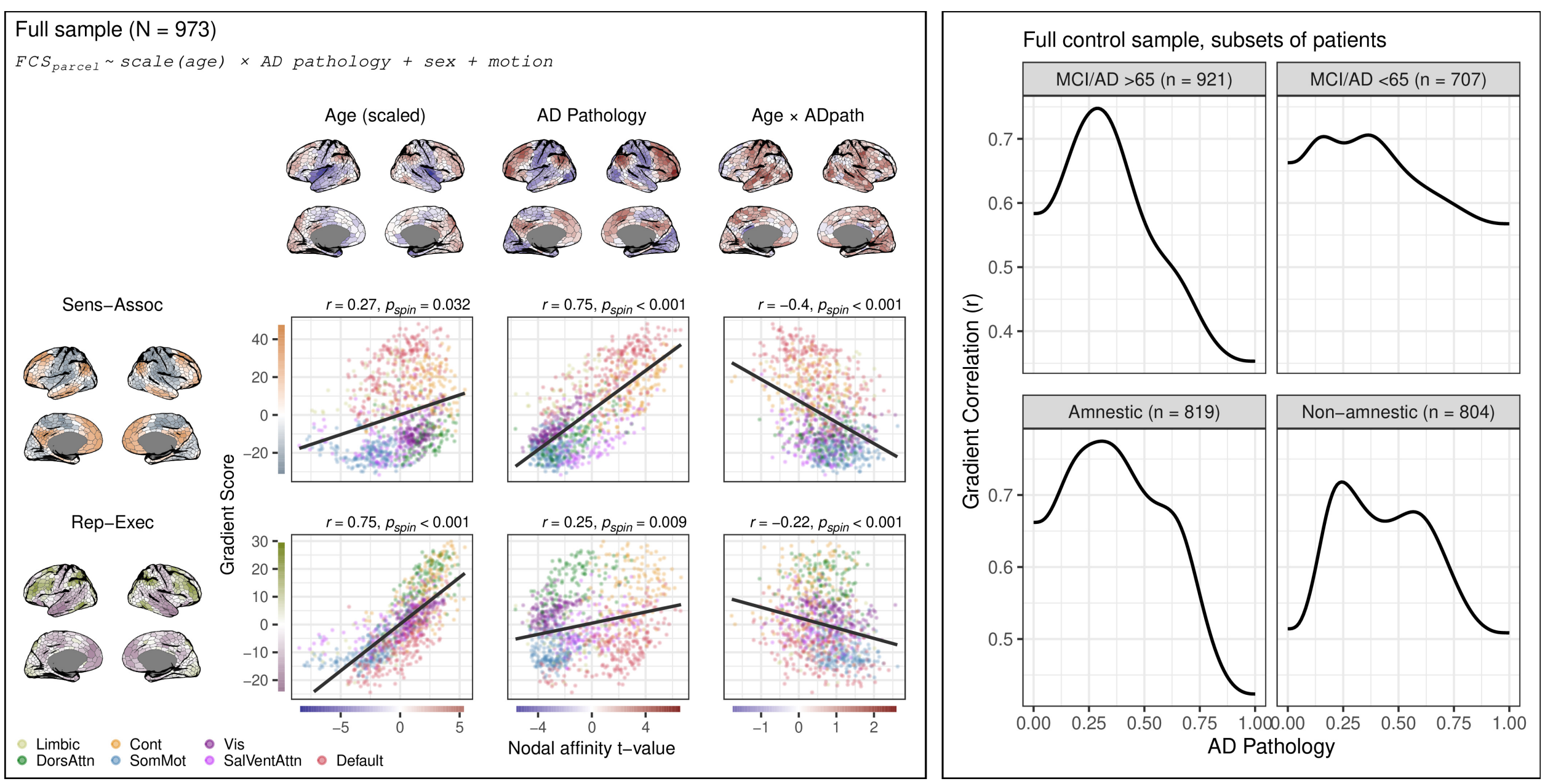

Supplementary Fig. 2: Age attenuates Alzheimer's disease–related gradient alignment, whereas cognitive phenotype shows limited modulation. Left: Parcel-wise linear regression results in the full sample (N = 973), modeling FCS as a function of age (scaled), AD pathology, and their interaction (controlling for sex and motion). Surface maps show t-values for the main effects and the age × pathology interaction. Scatterplots illustrate the correspondence between parcel-wise t-values and the sensory–association and representational–executive axes, with Pearson correlations and spin-test p-values indicated. The age × pathology interaction shows an inverse alignment with the SA axis, indicating attenuation of pathology-related gradient alignment at older ages. Right: Stratified nonlinear analyses of SA alignment across the AD pathology continuum. Using the full control sample but restricting patient subsets, gradient correlations with the first derivatives of the non-linear parcel-wise slopes were estimated across 100 pathology values from 0 to 1. In the top plot patients were stratified by age and in the bottom plot they were stratified as amnestic/non-amnestic by performance on cognitive domain scores. Domain scores were derived through a bifactor model of cognition visualised in Figure 6. Older patients show a steeper decline in SA axis alignment at higher pathology levels compared to younger patients, whereas both amnestic and

non-amnestic groups exhibit broadly similar trajectories. Together, these analyses indicate that age modulates the strength of pathology-related gradient alignment, while broad cognitive phenotype (within typical AD presentation) does not fully account for the observed loss of alignment at later disease stages.

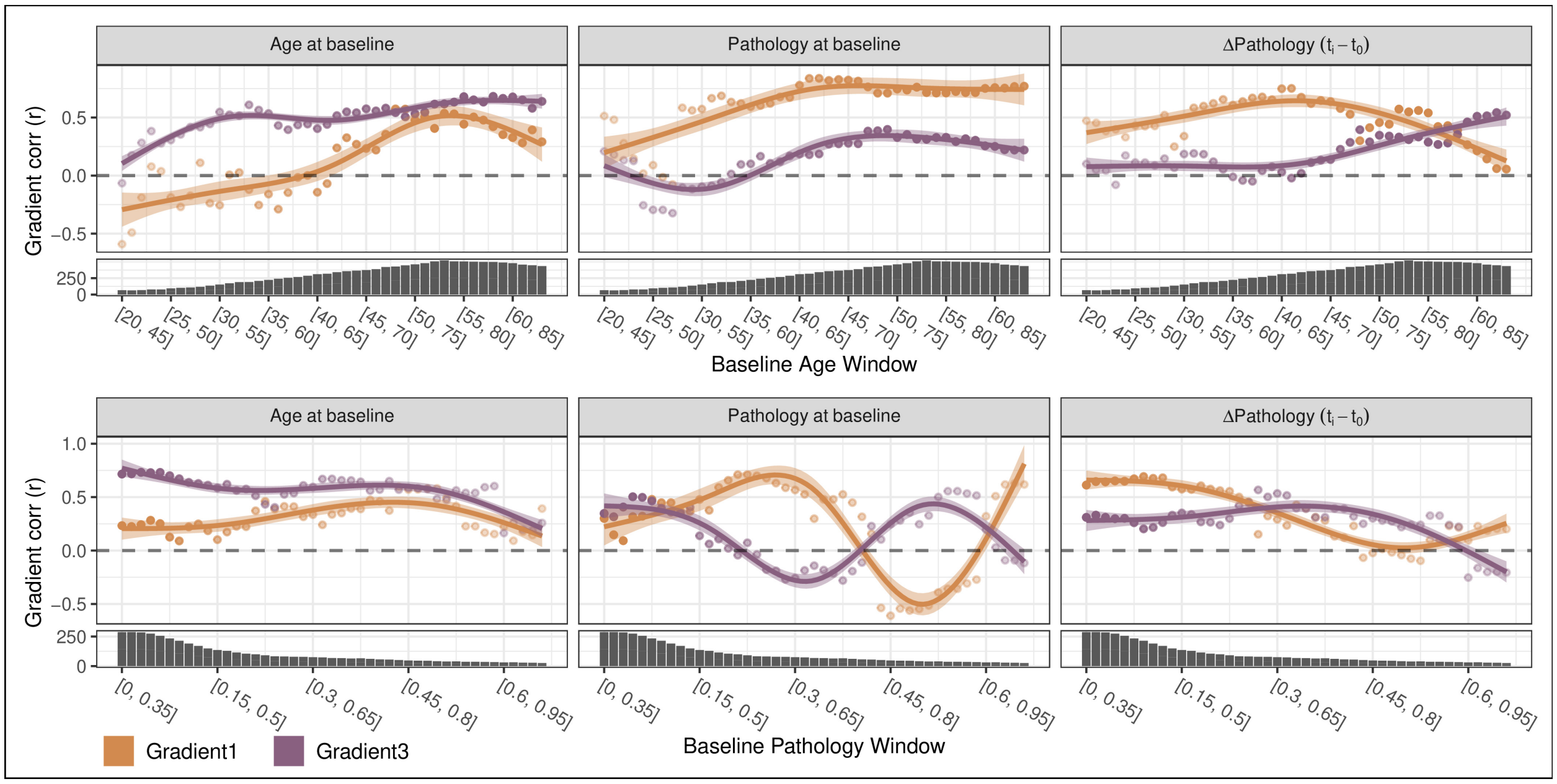

Supplementary Fig. 3: Results of the sliding window analysis from Figure 4 showing the dynamic effect of AD pathology on FC gradient alignment. The correlation values on the scatters are smoothed with generalized additive models. Correlations are shown for each term for both the SA and RE axis. Marginal plots show the sample size for each window. At higher baseline-pathology windows, pathology-related FC effects show a negative association with the sensory–association axis, coinciding with a transient rise in alignment with the representational–executive axis. This crossover indicates that the spatial organization of pathology-related FC changes may shift as pathology advances.

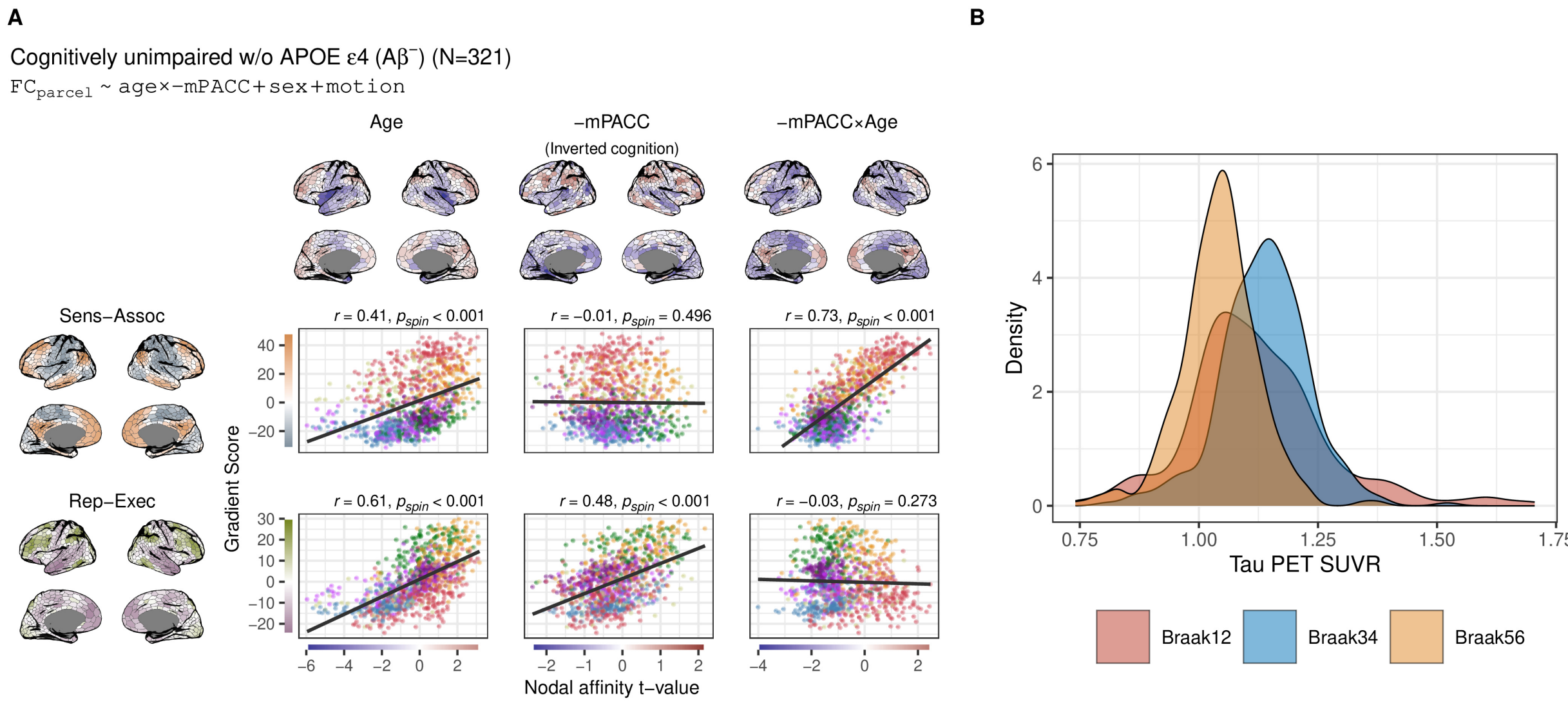

Supplementary Fig. 4: A: Cross-sectional results for cognitively unimpaired Aβ-, *APOE* ε4 non-carriers. The model includes age, inverted cognition (-mPACC), and an age × cognition interaction (with centered predictors and inverted cognition scores for interpretability). Unlike the main analysis presented in Figure 5, this analysis does not adjust for pathology. The age effects observed here partially reflect the effects previously attributed to pathology, revealing a relationship with the SA axis that diminishes when adjusting for pathology. Cortical maps display t-values from nodal linear regression models, while scatter plots show the relationships between the t-values and gradient scores, colored by network membership. The relationship was quantified using Pearson correlation and significance assessed using a spin test (one-sided, uncorrected; see Methods). B: Distribution of tau PET SUVR from Braak I-II, Braak III-IV and Braak V-VI in cognitively unimpaired Aβ-, *APOE* ε4 non-carriers.

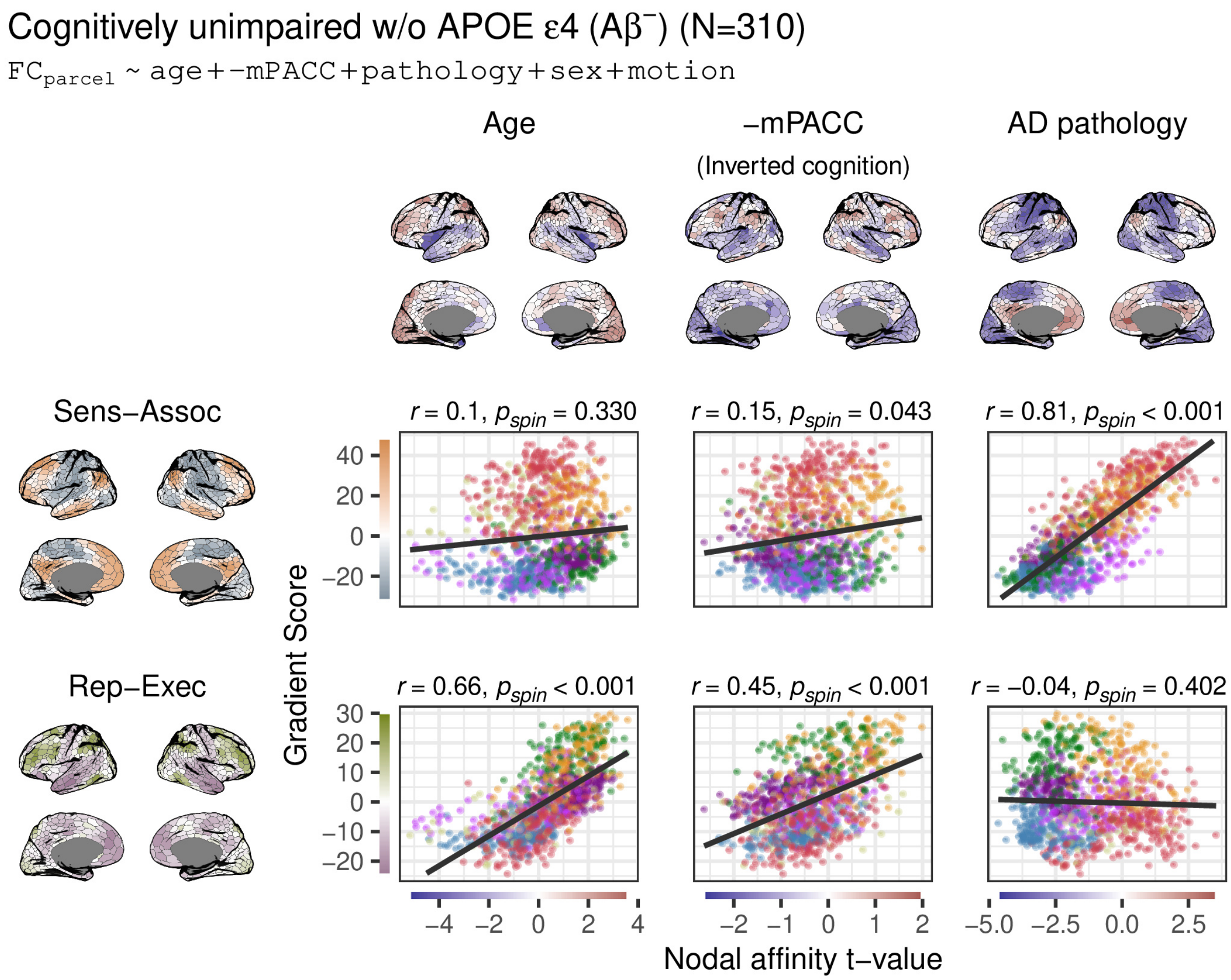

Supplementary Fig. 5: Cross-sectional results for cognitively unimpaired Aβ-, *APOE* ε4 non-carriers. This model is without interaction between cognition and age.

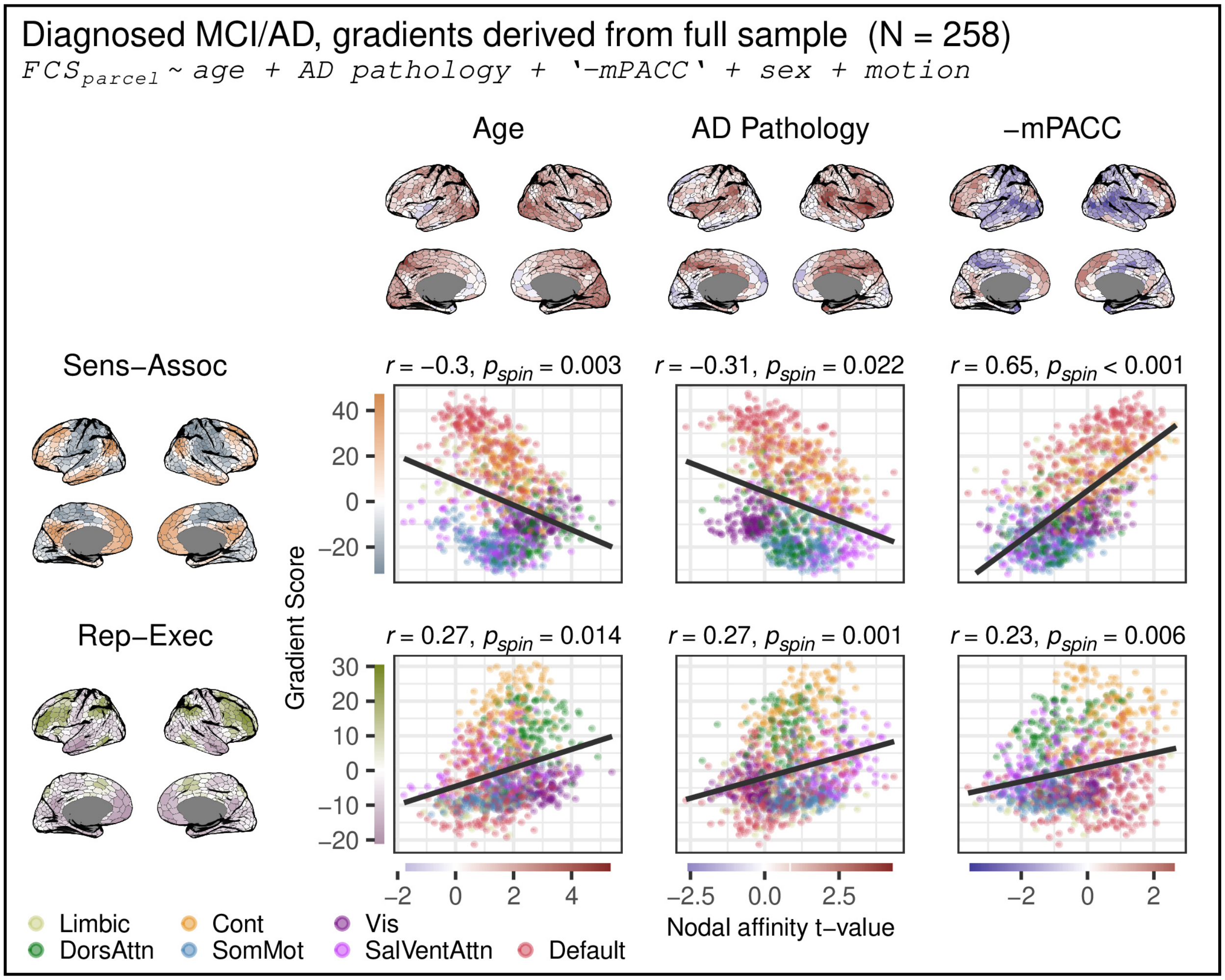

Supplementary Fig. 6: To investigate whether the lost association between AD pathology and the sensory association axis in the cognitively impaired subgroup analysis was related to a bias in deriving the gradients only from cognitively healthy subjects below the age of 60, we derived gradients from the full sample and ran the same analysis. This produced consistent results.

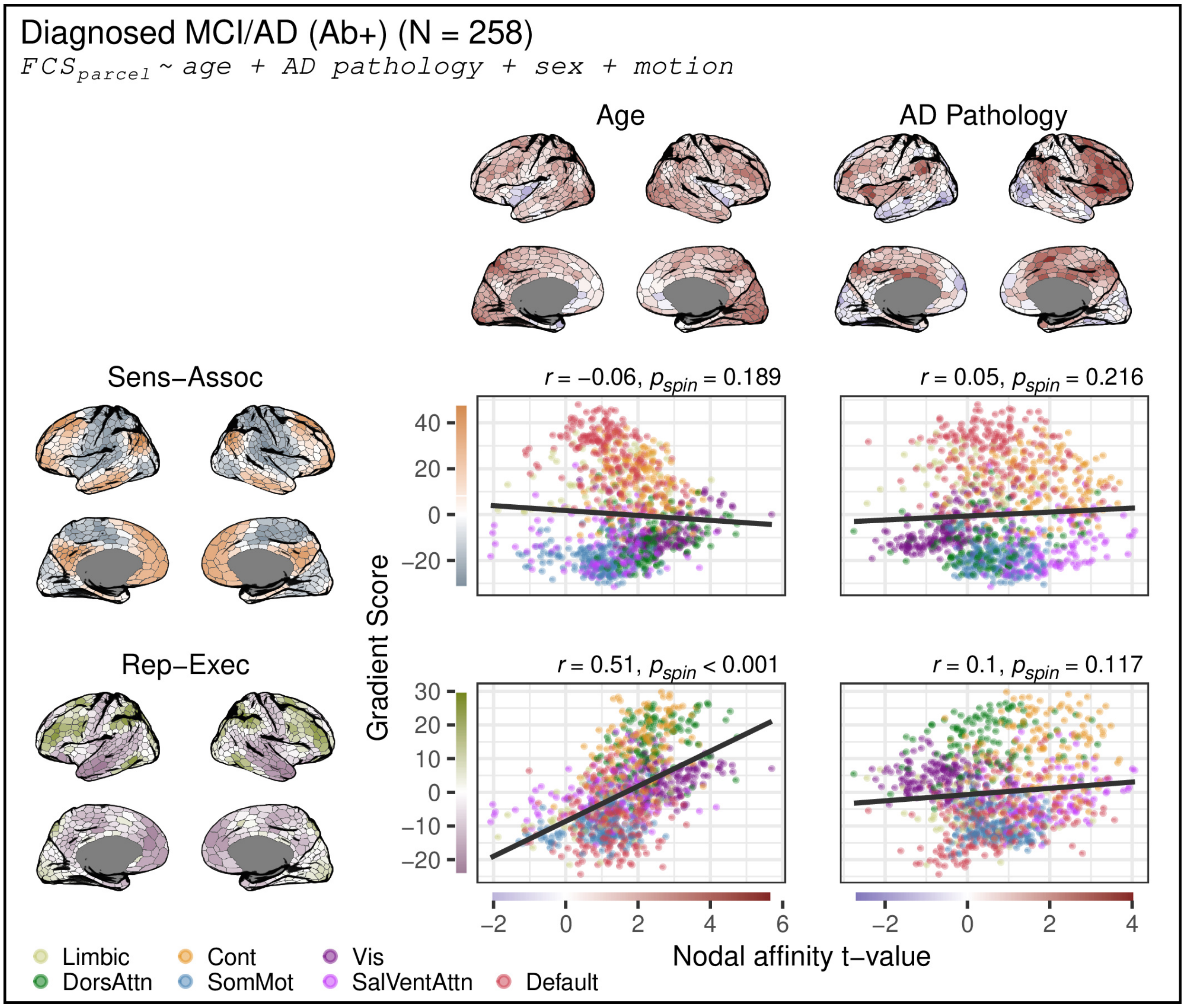

Supplementary Fig. 7: Cross-sectional results within the MCI and AD group without cognition. This analysis revealed that AD pathology showed no relationship to the SA axis even when not accounting for cognition, within this group.

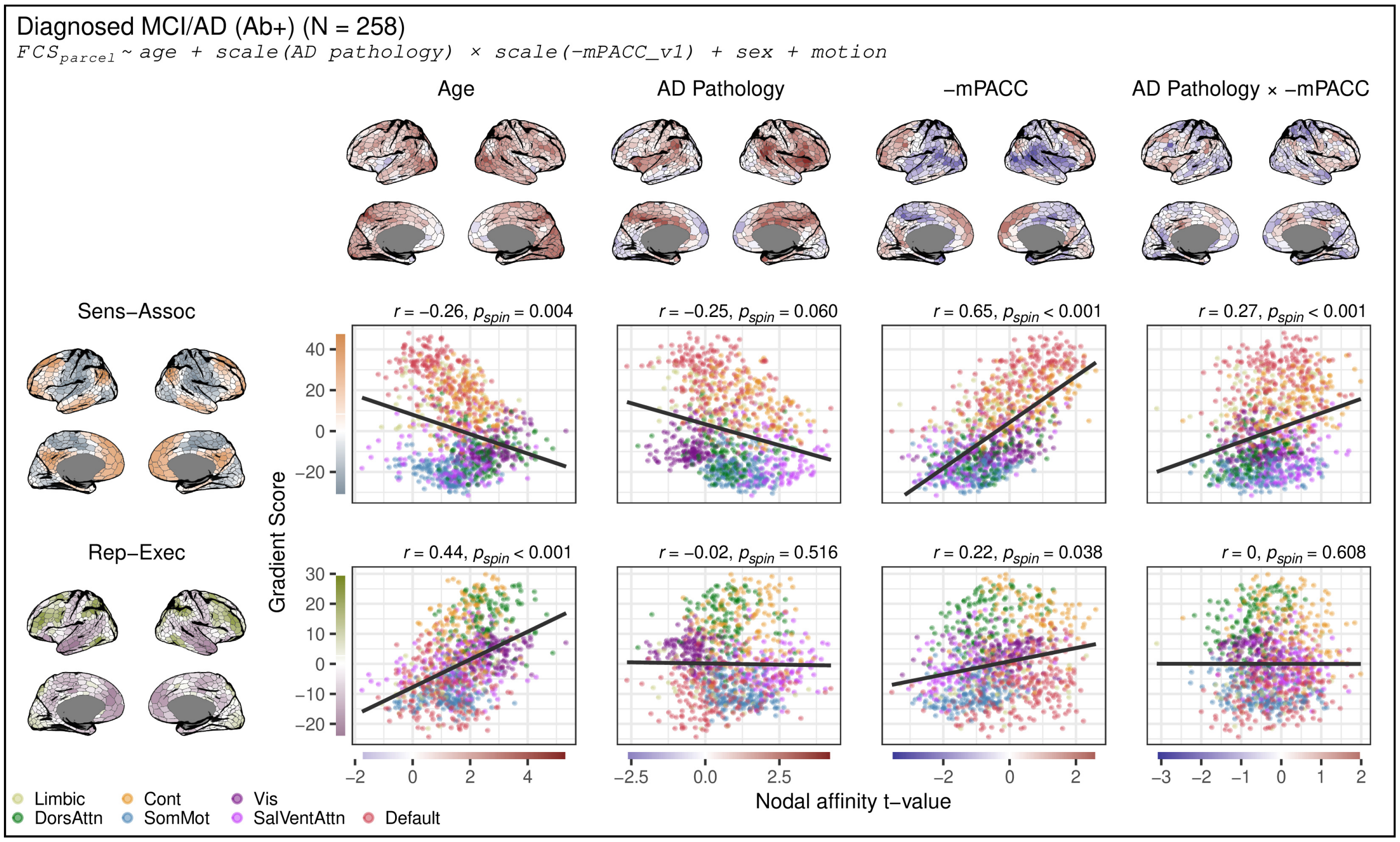

Supplementary Fig. 8: Cross-sectional analyses within patients with mild cognitive impairment (MCI) and Alzheimer's disease (AD), interacting AD pathology and cognition revealed that this did not affect the main effect of cognition. Predictors have been centred and cognition scores inverted for interpretability. Cortical maps display t-values from nodal linear regression models, while scatter plots show the relationships between the t-values and gradient scores, colored by network membership. The relationship was quantified using Pearson correlation and significance assessed using a spin test (one-sided, uncorrected; see Methods).

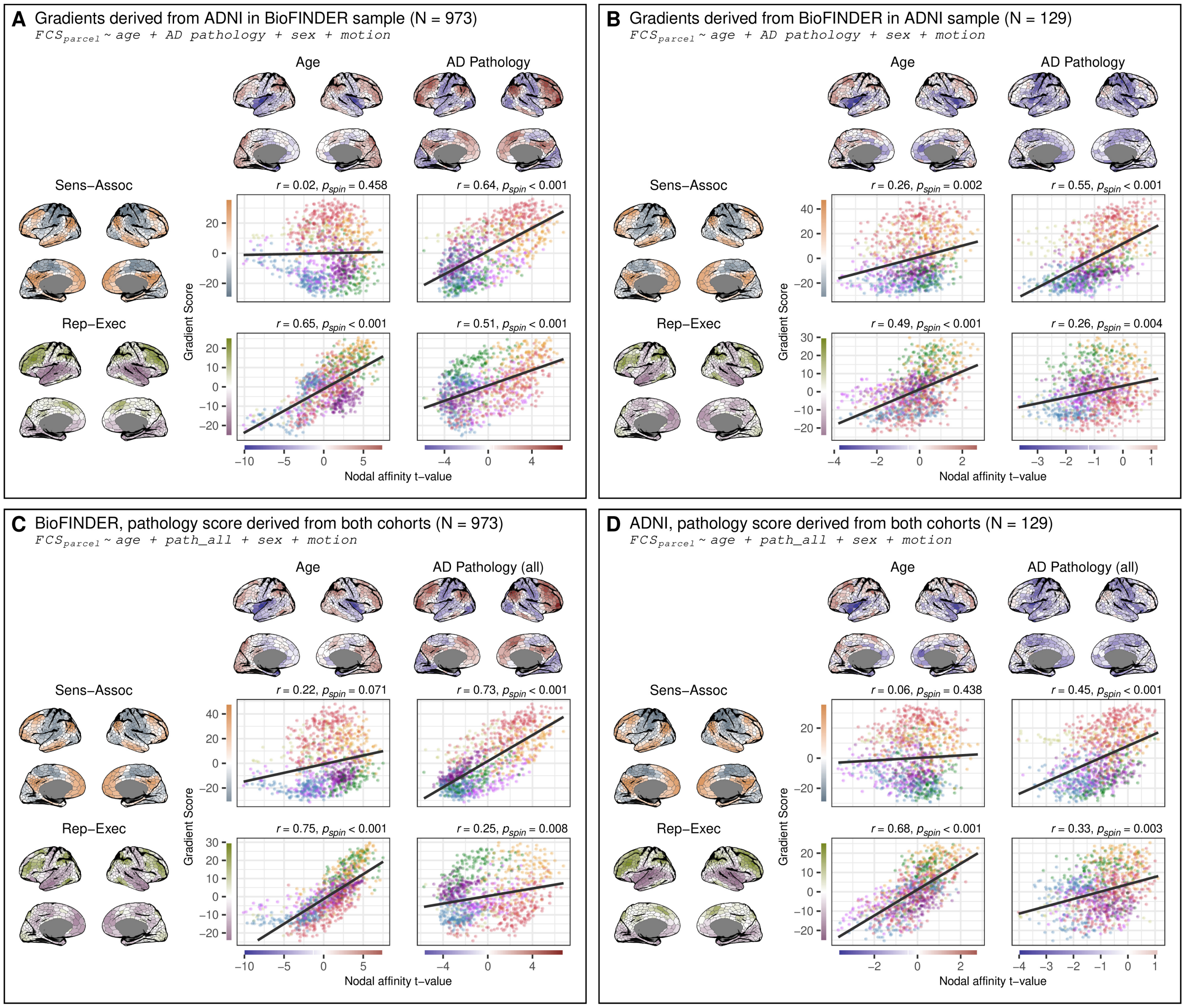

Supplementary Fig. 9: Main results but using gradients derived from ADNI to interpret the t-maps in BioFINDER (A) and the gradients derived from BioFINDER to interpret the t-maps from ADNI (B). To investigate whether the calculation pathology scores within each cohort affected the results we derived a pathology score after pooling data from the two samples and running the main analysis again in both BioFINDER (C) and ADNI (D). Results were comparable with the originally derived pathology score within each cohort.

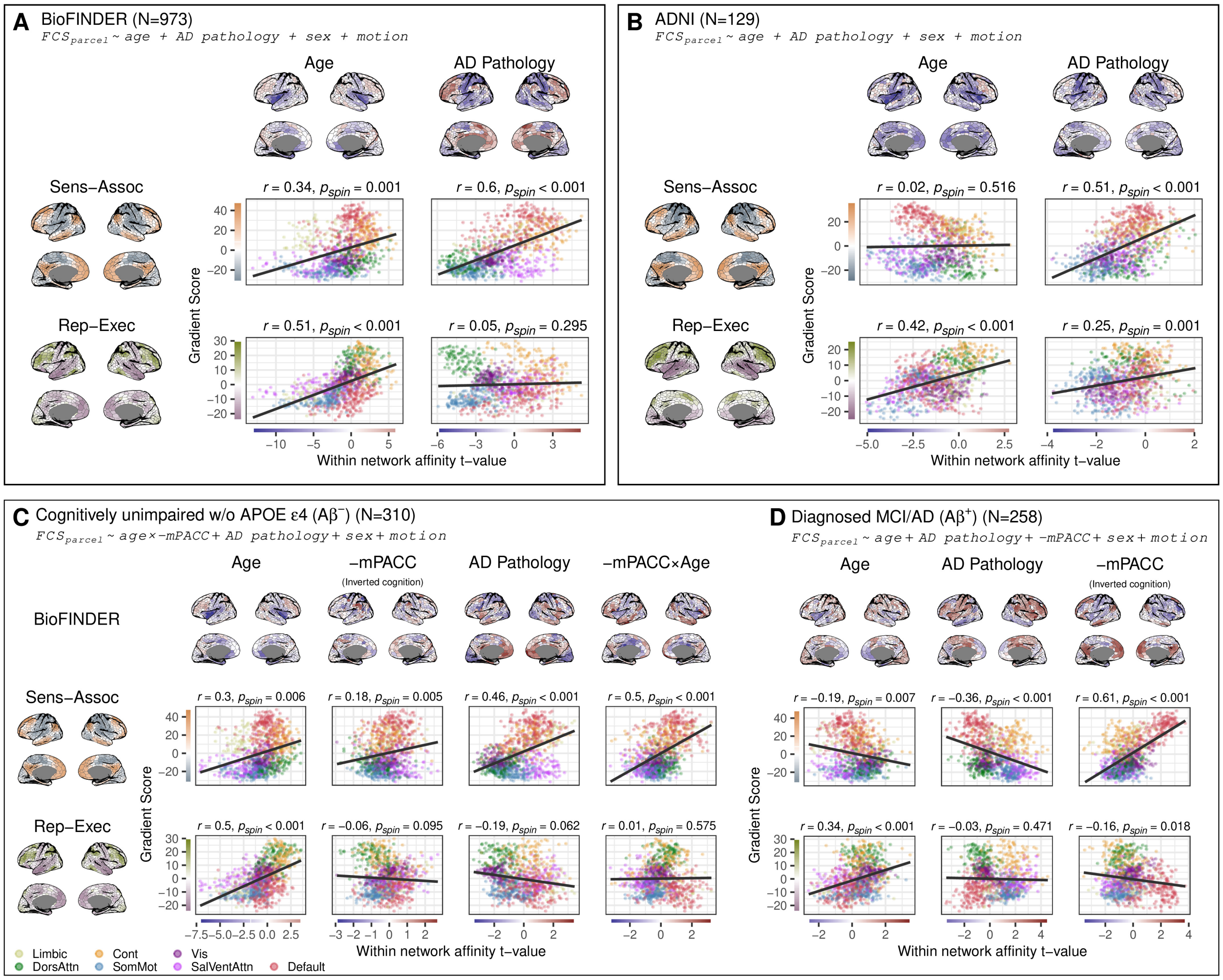

Supplementary Fig. 10: Analyses replicated with within-network affinity. Within-network affinity was calculated as the average affinity between parcels within the same Yeo network[2]. Results are shown for BioFINDER (A), ADNI (B). How cognitive status seems to modify these relationships are shown in (C) and (D). Cortical maps display t-values from nodal linear models, while scatter plots show the relationships between t-values and gradient scores, colored by network membership. The relationship was quantified using Pearson correlation and significance assessed using a spin test (one-sided, uncorrected; see Methods).

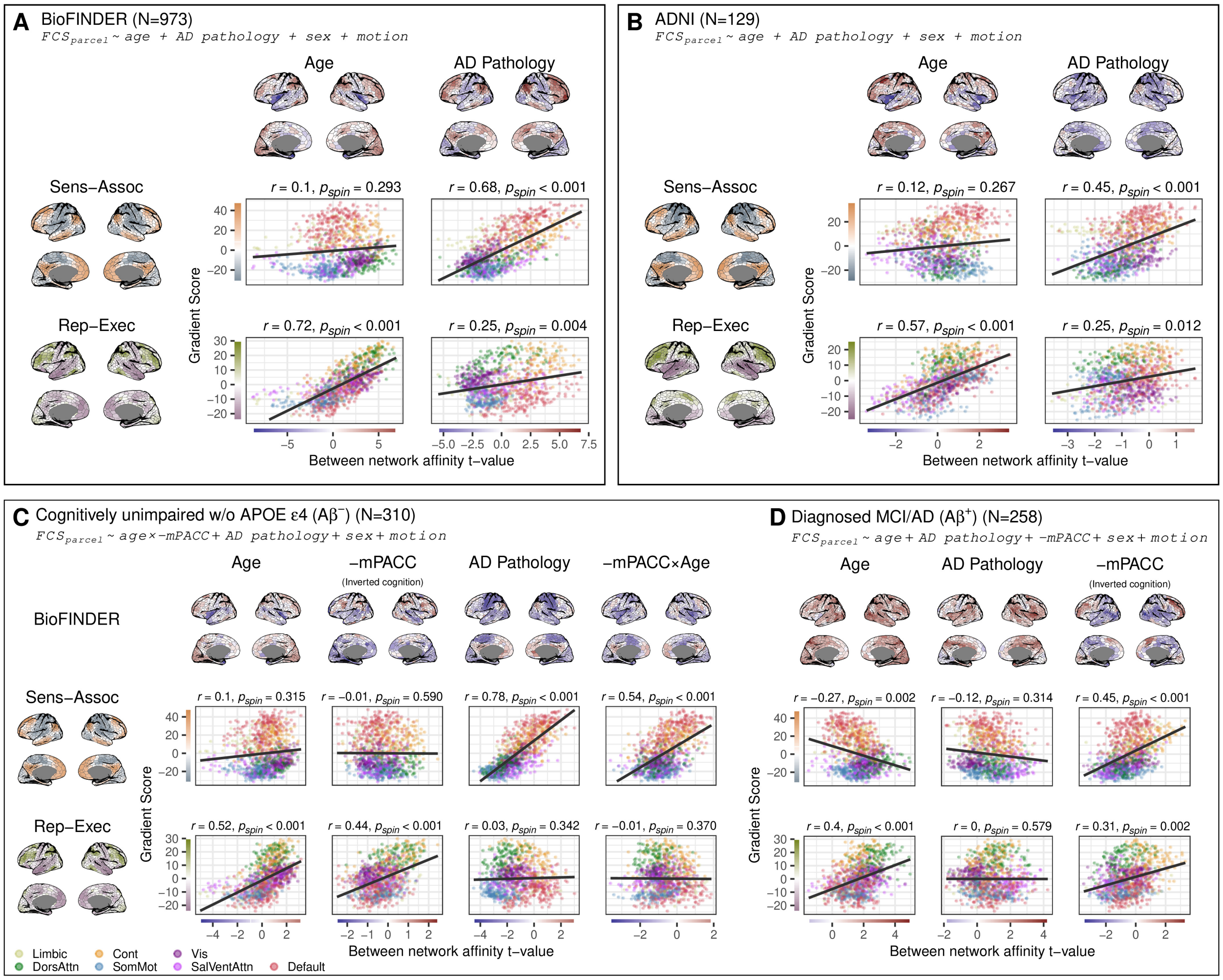

Supplementary Fig. 11: Analyses replicated with between-network affinity. Between-network affinity was calculated as the average affinity to parcels outside of a parcel's own Yeo 7 network[2]. Results are shown for BioFINDER (A), ADNI (B). How cognitive status seems to modify these relationships are shown in (C) and (D). Cortical maps display t-values from nodal linear models, while scatter plots show the relationships between t-values and gradient scores, colored by network membership. The relationship was quantified using Pearson correlation and significance assessed using a spin test (one-sided, uncorrected; see Methods).

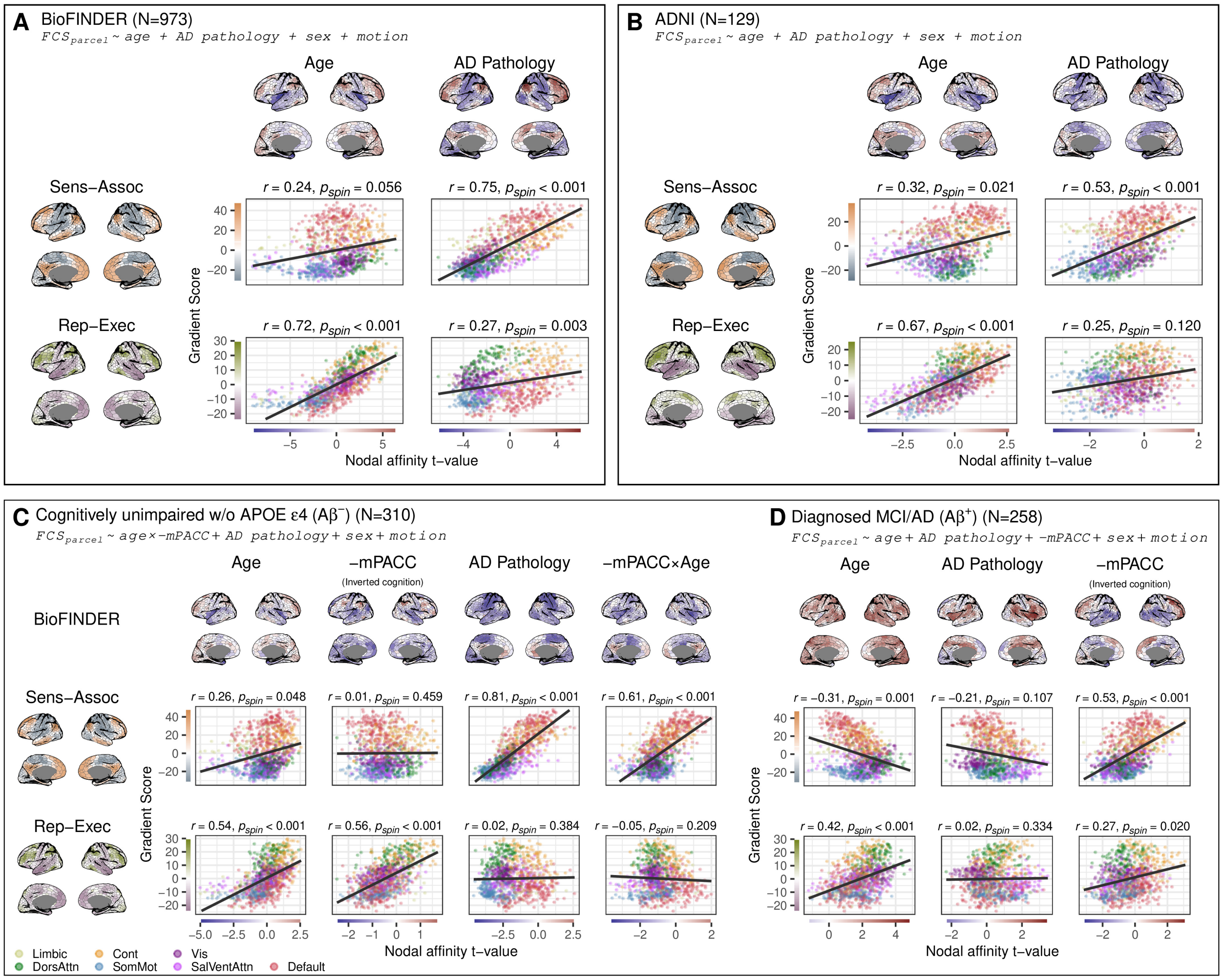

Supplementary Fig. 12: Analyses replicated with nodal affinity calculated using Pearson correlation instead of cosine similarity and no thresholding demonstrate consistent results with the main analyses (Figure 2 and Figure 5). Results are shown for BioFINDER (A), ADNI (B). How cognitive status seems to modify these relationships are shown in (C) and (D). Cortical maps display t-values from nodal linear models, while scatter plots show the relationships between t-values and gradient scores, colored by network membership. The relationship was quantified using Pearson correlation and significance assessed using a spin test (one-sided, uncorrected; see Methods).

1. Margulies, D. S. *et al.* Situating the default-mode network along a principal gradient of macroscale cortical organization. *Proceedings of the National Academy of Sciences* **113**, 12574–12579 (2016).

2. Yeo, B. T. *et al.* The organization of the human cerebral cortex estimated by intrinsic functional connectivity. *Journal of Neurophysiology* **106**, 1125–1165 (2011).
