# Supplementary material for: Different functional connectivity gradients reflect aging and Alzheimer’s disease
Source: Nat Neurosci. 2026 Oct 1;29(11):2835–47. doi: 10.1038/s41593-026-02402-0 (PMC13724280; doi:10.1038/s41593-026-02402-0)
Supplement: Supplementary file 2 [file 41593_2026_2402_MOESM2_ESM.pdf]

nature portfolio

Corresponding author(s): Jonathan Rittmo; Jacob W Vogel

Last updated by author(s): 02/05/26

Reporting Summary

Nature Portfolio wishes to improve the reproducibility of the work that we publish. This form provides structure for consistency and transparency in reporting. For further information on Nature Portfolio policies, see our Editorial Policies and the Editorial Policy Checklist.

Statistics

For all statistical analyses, confirm that the following items are present in the figure legend, table legend, main text, or Methods section.

n/a	Confirmed	
☐	☒	The exact sample size (*n*) for each experimental group/condition, given as a discrete number and unit of measurement
☐	☒	A statement on whether measurements were taken from distinct samples or whether the same sample was measured repeatedly
☐	☒	The statistical test(s) used AND whether they are one- or two-sided<br>*Only common tests should be described solely by name; describe more complex techniques in the Methods section.*
☐	☒	A description of all covariates tested
☐	☒	A description of any assumptions or corrections, such as tests of normality and adjustment for multiple comparisons
☐	☒	A full description of the statistical parameters including central tendency (e.g. means) or other basic estimates (e.g. regression coefficient) AND variation (e.g. standard deviation) or associated estimates of uncertainty (e.g. confidence intervals)
☐	☒	For null hypothesis testing, the test statistic (e.g. *F*, *t*, *r*) with confidence intervals, effect sizes, degrees of freedom and *P* value noted<br>*Give P values as exact values whenever suitable.*
☒	☐	For Bayesian analysis, information on the choice of priors and Markov chain Monte Carlo settings
☒	☐	For hierarchical and complex designs, identification of the appropriate level for tests and full reporting of outcomes
☐	☒	Estimates of effect sizes (e.g. Cohen's *d*, Pearson's *r*), indicating how they were calculated

Our web collection on statistics for biologists contains articles on many of the points above.

Software and code

Policy information about availability of computer code

Data collection	No software was used to collect the data in this study.
Data analysis	Preprocessing of the biofinder fMRI data was performed using a modified CPAC12 pipeline, building mostly on FSL, AFNI and ANTS. Skullstripping was done with in-house code using the T2 structural image as a primer and Brain Surface Extractor from BrainSuite. The fMRI preprocessing included slice-timing and motion correction. Physiological noise was regressed out using CompCor, alongside the removal of linear and quadratic trends. Additionally, regression of Friston's 24-parameter motion correction18, white matter and CSF signals were performed. Susceptibility distortion was corrected by unwarping the functional data using a nonlinear diffeomorphic transformation (performed with ANTS) of the mean functional image to high resolution (1 × 1 × 1 mm3) T2 structural image19. This transformation was then applied to each volume in the fMRI timeseries. Finally, a bandpass filter (0.01-0.1 Hz) was applied, and images were transformed from native to MNI space with ANTS.<br><br>Timeseries extraction from fMRI Nifti images was performed using Nilearn and meta-analytic decoding was performed using NiMARE (0.6.0) in Python 3.12, neuro, all other analyses and visualisation was done using R 4.4.2 (2024) using an array of different packages including: boot (1.3-32), broom (1.0.8), clue (0.3-66), confintr (1.0.2), conflicted (1.2.0), cowplot (1.1.3), dplyr (1.1.4), finalfit (1.0.8), flextable (0.9.7), freesurferformats (1.0.0), gganimate (1.0.11), ggforce (0.5.0), ggnewscale (0.5.1), ggplot2 (3.5.2), ggpmisc (0.6.1), ggpp (0.5.8-1), ggpubr (0.6.0), ggraph (2.2.2), ggrastr (1.0.2), ggsci (3.2.0), ggside (0.3.1), ggtext (0.1.2), ggwordcloud (0.6.2), gratia (0.10.0), gtable (0.3.6), httr2 (1.2.1), janitor (2.2.1), jsonlite (2.0.0), kableExtra (1.4.0), knitr (1.50), lme4 (1.1-37), magick (2.8.6), matrixStats (1.5.0), mediation (4.5.1), mice (3.17.0), mixtools (2.0.0.1), patchwork (1.3.0), pbapply (1.7-2), pbmcapply (1.5.1), performance (0.15.2), plotGMM (0.2.2), png (0.1-8), ppcor (1.1), proxy (0.4-27), proxyC (0.5.2), psych (2.5.6), purrr (1.0.4), quarto (1.5.1), ragg (1.4.0), RColorBrewer (1.1-3), readr (2.1.5), readxl (1.4.5), renv (1.0.7), reticulate (1.42.0), rlang (1.1.6), rmarkdown (2.29), RSpectra (0.16-2), scales (1.4.0), SCORPIUS (1.0.9), sf (1.1-0), stringdist (0.9.15), stringr (1.5.1), tibble (3.2.1), tidygraph (1.3.1), tidyr (1.3.1), tidyverse (2.0.0), withr (3.0.2), base (4.5.3), grid (4.5.3), Matrix (1.7-4),

mgcv (1.9-4), parallel (4.5.3), stats (4.5.3), tools (4.5.3), utils (4.5.3). Code for all analyses and visualisation is available at https://github.com/DeMONLab-BioFINDER/fc_changes_follow_gradients.

For manuscripts utilizing custom algorithms or software that are central to the research but not yet described in published literature, software must be made available to editors and reviewers. We strongly encourage code deposition in a community repository (e.g. GitHub). See the Nature Portfolio guidelines for submitting code & software for further information.

Data

Policy information about availability of data

All manuscripts must include a data availability statement. This statement should provide the following information, where applicable:

- Accession codes, unique identifiers, or web links for publicly available datasets
- A description of any restrictions on data availability
- For clinical datasets or third party data, please ensure that the statement adheres to our policy

Data from the validation cohort ADNI is a public access dataset and can be obtained by application from http://adni.loni.usc.edu/. The BioFINDER data are not publicly available, but anonymized data may be made available upon request to qualified academic investigators. Data sharing must comply with EU General Data Protection Regulation (GDPR), decisions of the Swedish Ethical Review Authority, and regulations of Region Skåne.

Research involving human participants, their data, or biological material

Policy information about studies with human participants or human data. See also policy information about sex, gender (identity/presentation), and sexual orientation and race, ethnicity and racism.

Reporting on sex and gender	Sex was self reported and used as a covariate in all analyses. The distribution of sexes within disease groups can be found in population characteristics (Table 1).
Reporting on race, ethnicity, or other socially relevant groupings	Our study did not explicitly use race, ethnicity, or other socially constructed/relevant categorization variables in any analyses. Such variables were not collected in the BioFINDER study. However, BioFINDER includes data on participants' native language, and among those included in our primary cross-sectional analysis that had this data available (~96%), 94% reported Swedish as their native language. The ADNI study does include self-reported race, with 94% of our analyzed sample identifying as White; however, we did not explicitly utilize race data in any statistical analyses or interpretations, nor did we use these variables as proxies. Nevertheless, we acknowledge that recruitment from predominantly White (ADNI) and Swedish-speaking (BioFINDER) populations implicitly introduces a demographic bias that may limit the generalizability of our findings. This is mentioned in the limitations of the article.
Population characteristics	Information population characteristics can be found at https://biofinder.se/two/population-study-design. But for the cross-sectional sample from BioFINDER in this study, characteristics included 973 individuals: 655 cognitively normal (CN), 152 with Aβ-positive mild cognitive impairment (MCI Aβ+), and 166 with Alzheimer's disease (AD). CN participants were younger (mean age 63.4) compared to MCI Aβ+ and AD groups (both ~72.5 years), and showed lower pathology scores and SUVRs across Braak regions. Aβ positivity was present in all MCI Aβ+ and AD participants, but only 27.6% of the CN group. Functional MRI head motion (mean FD) and years of education were similar across groups. The CN group had a higher proportion of women (54.5%), while the MCI Aβ+ group had more men (57.2%), and the AD group had somewhat more women (53%).
Recruitment	Information regarding recruitment criteria can be found at https://biofinder.se/two/population-study-design/
Ethics oversight	Ethical approval for BioFINDER-2 was obtained from the Ethics Committee of Lund University, Lund, Sweden, with additional permissions for PET imaging granted by the Swedish Medicines and Products Agency and the local Radiation Safety Committee at Skåne University Hospital, Sweden. For ADNI, ethical approval was obtained by the ADNI investigators, all participants provided written informed consent.

Note that full information on the approval of the study protocol must also be provided in the manuscript.

Field-specific reporting

Please select the one below that is the best fit for your research. If you are not sure, read the appropriate sections before making your selection.

☒ Life sciences ☐ Behavioural & social sciences ☐ Ecological, evolutionary & environmental sciences

For a reference copy of the document with all sections, see nature.com/documents/nr-reporting-summary-flat.pdf

Life sciences study design

All studies must disclose on these points even when the disclosure is negative.

Sample size	There were several different and overlapping samples used in the present study. The main cross-sectional cohort, BioFINDER-2 had n = 973. The validation cohort ADNI had n = 129. The longitudinal subsample in Biofinder had n = 378. Sub-group analyses in cognitively healthy without AD biomarker positivity, n = 310, and in diagnosed MCI/AD, n = 258.
Data exclusions	Individuals with too high scanner motion were excluded. MCI and AD patients were only included if they were also amyloid-beta positive. And individuals with known neurodegenerative diseases other than Alzheimer's were excluded.

Replication	The main analysis was replicated in an independent cohort (ADNI), and was replicated longitudinally within the discover cohort.
Randomization	Randomization was not performed as the study is observational.
Blinding	Data from BioFINDER-2 was processed blinded to participant diagnosis and biomarker status

Behavioural & social sciences study design

All studies must disclose on these points even when the disclosure is negative.

Study description	*Briefly describe the study type including whether data are quantitative, qualitative, or mixed-methods (e.g. qualitative cross-sectional, quantitative experimental, mixed-methods case study).*
Research sample	*State the research sample (e.g. Harvard university undergraduates, villagers in rural India) and provide relevant demographic information (e.g. age, sex) and indicate whether the sample is representative. Provide a rationale for the study sample chosen. For studies involving existing datasets, please describe the dataset and source.*
Sampling strategy	*Describe the sampling procedure (e.g. random, snowball, stratified, convenience). Describe the statistical methods that were used to predetermine sample size OR if no sample-size calculation was performed, describe how sample sizes were chosen and provide a rationale for why these sample sizes are sufficient. For qualitative data, please indicate whether data saturation was considered, and what criteria were used to decide that no further sampling was needed.*
Data collection	*Provide details about the data collection procedure, including the instruments or devices used to record the data (e.g. pen and paper, computer, eye tracker, video or audio equipment) whether anyone was present besides the participant(s) and the researcher, and whether the researcher was blind to experimental condition and/or the study hypothesis during data collection.*
Timing	*Indicate the start and stop dates of data collection. If there is a gap between collection periods, state the dates for each sample cohort.*
Data exclusions	*If no data were excluded from the analyses, state so OR if data were excluded, provide the exact number of exclusions and the rationale behind them, indicating whether exclusion criteria were pre-established.*
Non-participation	*State how many participants dropped out/declined participation and the reason(s) given OR provide response rate OR state that no participants dropped out/declined participation.*
Randomization	*If participants were not allocated into experimental groups, state so OR describe how participants were allocated to groups, and if allocation was not random, describe how covariates were controlled.*

Ecological, evolutionary & environmental sciences study design

All studies must disclose on these points even when the disclosure is negative.

Study description	*Briefly describe the study. For quantitative data include treatment factors and interactions, design structure (e.g. factorial, nested, hierarchical), nature and number of experimental units and replicates.*
Research sample	*Describe the research sample (e.g. a group of tagged Passer domesticus, all Stenocereus thurberi within Organ Pipe Cactus National Monument), and provide a rationale for the sample choice. When relevant, describe the organism taxa, source, sex, age range and any manipulations. State what population the sample is meant to represent when applicable. For studies involving existing datasets, describe the data and its source.*
Sampling strategy	*Note the sampling procedure. Describe the statistical methods that were used to predetermine sample size OR if no sample-size calculation was performed, describe how sample sizes were chosen and provide a rationale for why these sample sizes are sufficient.*
Data collection	*Describe the data collection procedure, including who recorded the data and how.*
Timing and spatial scale	*Indicate the start and stop dates of data collection, noting the frequency and periodicity of sampling and providing a rationale for these choices. If there is a gap between collection periods, state the dates for each sample cohort. Specify the spatial scale from which the data are taken*
Data exclusions	*If no data were excluded from the analyses, state so OR if data were excluded, describe the exclusions and the rationale behind them, indicating whether exclusion criteria were pre-established.*
Reproducibility	*Describe the measures taken to verify the reproducibility of experimental findings. For each experiment, note whether any attempts to repeat the experiment failed OR state that all attempts to repeat the experiment were successful.*
Randomization	*Describe how samples/organisms/participants were allocated into groups. If allocation was not random, describe how covariates were controlled. If this is not relevant to your study, explain why.*

Blinding	*Describe the extent of blinding used during data acquisition and analysis. If blinding was not possible, describe why OR explain why blinding was not relevant to your study.*

Did the study involve field work? ☐ Yes ☐ No

Field work, collection and transport

Field conditions	*Describe the study conditions for field work, providing relevant parameters (e.g. temperature, rainfall).*
Location	*State the location of the sampling or experiment, providing relevant parameters (e.g. latitude and longitude, elevation, water depth).*
Access & import/export	*Describe the efforts you have made to access habitats and to collect and import/export your samples in a responsible manner and in compliance with local, national and international laws, noting any permits that were obtained (give the name of the issuing authority, the date of issue, and any identifying information).*
Disturbance	*Describe any disturbance caused by the study and how it was minimized.*

Reporting for specific materials, systems and methods

We require information from authors about some types of materials, experimental systems and methods used in many studies. Here, indicate whether each material, system or method listed is relevant to your study. If you are not sure if a list item applies to your research, read the appropriate section before selecting a response.

Materials & experimental systems

n/a	Involved in the study	
☒	☐	Antibodies
☒	☐	Eukaryotic cell lines
☒	☐	Palaeontology and archaeology
☒	☐	Animals and other organisms
☐	☒	Clinical data
☒	☐	Dual use research of concern
☒	☐	Plants

Methods

n/a	Involved in the study	
☒	☐	ChIP-seq
☒	☐	Flow cytometry
☐	☒	MRI-based neuroimaging

Antibodies

Antibodies used	*Describe all antibodies used in the study; as applicable, provide supplier name, catalog number, clone name, and lot number.*
Validation	*Describe the validation of each primary antibody for the species and application, noting any validation statements on the manufacturer's website, relevant citations, antibody profiles in online databases, or data provided in the manuscript.*

Eukaryotic cell lines

Policy information about cell lines and Sex and Gender in Research

Cell line source(s)	*State the source of each cell line used and the sex of all primary cell lines and cells derived from human participants or vertebrate models.*
Authentication	*Describe the authentication procedures for each cell line used OR declare that none of the cell lines used were authenticated.*
Mycoplasma contamination	*Confirm that all cell lines tested negative for mycoplasma contamination OR describe the results of the testing for mycoplasma contamination OR declare that the cell lines were not tested for mycoplasma contamination.*
Commonly misidentified lines (See ICLAC register)	*Name any commonly misidentified cell lines used in the study and provide a rationale for their use.*

Palaeontology and Archaeology

Specimen provenance	*Provide provenance information for specimens and describe permits that were obtained for the work (including the name of the issuing authority, the date of issue, and any identifying information). Permits should encompass collection and, where applicable, export.*

Specimen deposition | *Indicate where the specimens have been deposited to permit free access by other researchers.*

Dating methods | *If new dates are provided, describe how they were obtained (e.g. collection, storage, sample pretreatment and measurement), where they were obtained (i.e. lab name), the calibration program and the protocol for quality assurance OR state that no new dates are provided.*

☐ Tick this box to confirm that the raw and calibrated dates are available in the paper or in Supplementary Information.

Ethics oversight | *Identify the organization(s) that approved or provided guidance on the study protocol, OR state that no ethical approval or guidance was required and explain why not.*

Note that full information on the approval of the study protocol must also be provided in the manuscript.

Animals and other research organisms

Policy information about studies involving animals; ARRIVE guidelines recommended for reporting animal research, and Sex and Gender in Research

Laboratory animals | *For laboratory animals, report species, strain and age OR state that the study did not involve laboratory animals.*

Wild animals | *Provide details on animals observed in or captured in the field; report species and age where possible. Describe how animals were caught and transported and what happened to captive animals after the study (if killed, explain why and describe method; if released, say where and when) OR state that the study did not involve wild animals.*

Reporting on sex | *Indicate if findings apply to only one sex; describe whether sex was considered in study design, methods used for assigning sex. Provide data disaggregated for sex where this information has been collected in the source data as appropriate; provide overall numbers in this Reporting Summary. Please state if this information has not been collected. Report sex-based analyses where performed, justify reasons for lack of sex-based analysis.*

Field-collected samples | *For laboratory work with field-collected samples, describe all relevant parameters such as housing, maintenance, temperature, photoperiod and end-of-experiment protocol OR state that the study did not involve samples collected from the field.*

Ethics oversight | *Identify the organization(s) that approved or provided guidance on the study protocol, OR state that no ethical approval or guidance was required and explain why not.*

Note that full information on the approval of the study protocol must also be provided in the manuscript.

Clinical data

Policy information about clinical studies
All manuscripts should comply with the ICMJE guidelines for publication of clinical research and a completed CONSORT checklist must be included with all submissions.

Clinical trial registration | Registration numbers for BioFINDER-2: (ClinicalTrials.gov ID: NCT03174938); and ADNI (ClinicalTrials.gov ID: NCT02854033)

Study protocol | The BioFINDER study protocol and design is available at https://biofinder.se/two, for ADNI it is available at https://adni.loni.usc.edu/

Data collection | Information regarding collection are also available at https://biofinder.se/two, for ADNI it is available at https://adni.loni.usc.edu/

Outcomes | As this is an observational cohort study, no pre-specified clinical trial outcomes were defined. The primary outcomes in this study were measures of resting-state functional connectivity similarity derived from fMRI

Dual use research of concern

Policy information about dual use research of concern

Hazards

Could the accidental, deliberate or reckless misuse of agents or technologies generated in the work, or the application of information presented in the manuscript, pose a threat to:

No	Yes	
☒	☐	Public health
☒	☐	National security
☒	☐	Crops and/or livestock
☒	☐	Ecosystems
☒	☐	Any other significant area

Experiments of concern

Does the work involve any of these experiments of concern:

No	Yes	
☒	☐	Demonstrate how to render a vaccine ineffective
☒	☐	Confer resistance to therapeutically useful antibiotics or antiviral agents
☒	☐	Enhance the virulence of a pathogen or render a nonpathogen virulent
☒	☐	Increase transmissibility of a pathogen
☒	☐	Alter the host range of a pathogen
☒	☐	Enable evasion of diagnostic/detection modalities
☒	☐	Enable the weaponization of a biological agent or toxin
☒	☐	Any other potentially harmful combination of experiments and agents

Plants

Seed stocks	*Report on the source of all seed stocks or other plant material used. If applicable, state the seed stock centre and catalogue number. If plant specimens were collected from the field, describe the collection location, date and sampling procedures.*
Novel plant genotypes	*Describe the methods by which all novel plant genotypes were produced. This includes those generated by transgenic approaches, gene editing, chemical/radiation-based mutagenesis and hybridization. For transgenic lines, describe the transformation method, the number of independent lines analyzed and the generation upon which experiments were performed. For gene-edited lines, describe the editor used, the endogenous sequence targeted for editing, the targeting guide RNA sequence (if applicable) and how the editor was applied.*
Authentication	*Describe any authentication procedures for each seed stock used or novel genotype generated. Describe any experiments used to assess the effect of a mutation and, where applicable, how potential secondary effects (e.g. second site T-DNA insertions, mosiacism, off-target gene editing) were examined.*

ChIP-seq

Data deposition

☐ Confirm that both raw and final processed data have been deposited in a public database such as GEO.

☐ Confirm that you have deposited or provided access to graph files (e.g. BED files) for the called peaks.

Data access links<br>*May remain private before publication.*	*For "Initial submission" or "Revised version" documents, provide reviewer access links. For your "Final submission" document, provide a link to the deposited data.*
Files in database submission	*Provide a list of all files available in the database submission.*
Genome browser session<br>(e.g. UCSC)	*Provide a link to an anonymized genome browser session for "Initial submission" and "Revised version" documents only, to enable peer review. Write "no longer applicable" for "Final submission" documents.*

Methodology

Replicates	*Describe the experimental replicates, specifying number, type and replicate agreement.*
Sequencing depth	*Describe the sequencing depth for each experiment, providing the total number of reads, uniquely mapped reads, length of reads and whether they were paired- or single-end.*
Antibodies	*Describe the antibodies used for the ChIP-seq experiments; as applicable, provide supplier name, catalog number, clone name, and lot number.*
Peak calling parameters	*Specify the command line program and parameters used for read mapping and peak calling, including the ChIP, control and index files used.*
Data quality	*Describe the methods used to ensure data quality in full detail, including how many peaks are at FDR 5% and above 5-fold enrichment.*
Software	*Describe the software used to collect and analyze the ChIP-seq data. For custom code that has been deposited into a community repository, provide accession details.*

Flow Cytometry

Plots

Confirm that:

- [ ] The axis labels state the marker and fluorochrome used (e.g. CD4-FITC).
- [ ] The axis scales are clearly visible. Include numbers along axes only for bottom left plot of group (a 'group' is an analysis of identical markers).
- [ ] All plots are contour plots with outliers or pseudocolor plots.
- [ ] A numerical value for number of cells or percentage (with statistics) is provided.

Methodology

Sample preparation	*Describe the sample preparation, detailing the biological source of the cells and any tissue processing steps used.*
Instrument	*Identify the instrument used for data collection, specifying make and model number.*
Software	*Describe the software used to collect and analyze the flow cytometry data. For custom code that has been deposited into a community repository, provide accession details.*
Cell population abundance	*Describe the abundance of the relevant cell populations within post-sort fractions, providing details on the purity of the samples and how it was determined.*
Gating strategy	*Describe the gating strategy used for all relevant experiments, specifying the preliminary FSC/SSC gates of the starting cell population, indicating where boundaries between "positive" and "negative" staining cell populations are defined.*

- [ ] Tick this box to confirm that a figure exemplifying the gating strategy is provided in the Supplementary Information.

Magnetic resonance imaging

Experimental design

Design type	resting-state fMRI
Design specifications	rest
Behavioral performance measures	No performance measures were obtained in the scanner.

Acquisition

Imaging type(s)	Functional
Field strength	3T
Sequence & imaging parameters	BioFINDER. Structural MRI was acquired using a Siemens 3T MAGNETOM Prisma scanner (Siemens Healthineers, Erlangen, Germany) with a 64-channel head coil. T1-weighted images (Magnetization Prepared – Rapid Gradient Echo, MPRAGE) were acquired with the following parameters: in-plane resolution = 1 × 1 mm², slice thickness = 1 mm, repetition time (TR) = 1900 ms, echo time (TE) = 2.54 ms, flip-angle = 9°.<br>All resting-state fMRI data (eyes closed) were acquired using a 3D echo-planar imaging (EPI) sequence with an in-plane resolution of 3×3 mm and slice thickness 3.6 mm; echo time = 30ms, and flip-angle = 63°. Scan time was 7.85 minutes, with a multiband repetition time of 1020 ms, resulting in 462 frames per scan before processing and censoring.<br><br>ADNI. Briefly, MRI data were collected using 3T scanners with unified scanning protocols that can be accessed here: https://adni.loni.usc.edu/wp-content/uploads/2017/07/ADNI3-MRI-protocols.pdf. Structural MRI was acquired with a 3D T1-weighted MPRAGE sequence (1 mm isotropic voxels, TR = 2300 ms).<br>Two protocols exist for the resting state EPI-BOLD sequence in ADNI: Basic and Advanced. Depending on which protocol a participant has been subjected to, the parameters vary. Both protocols have an approximate scanner time of 10 minutes. The Basic protocol has a TR/TE/flip angle=3000/30/90° and the Advanced a TR/TE/flip angle=600/30/53°, so the number of frames differ depending on the protocol and ranges from approximately 200 to 1000.
Area of acquisition	Whole brain

Diffusion MRI: [ ] Used [x] Not used

Preprocessing

Preprocessing software	FSL, AFNI, ANTs and in-house scripts

Normalization

In both BioFINDER and ADNI normalization from native to MNI space was performed non-linearly using ANTs and T1-weighted images.

Normalization template

MNI152

Noise and artifact removal

Physiological noise was regressed out using CompCor, alongside the removal of linear and quadratic trends. Additionally, regression of Friston's 24-parameter motion correction, white matter and CSF signals were performed. Susceptibility distortion was corrected by unwarping the functional data using a nonlinear diffeomorphic transformation (performed with ANTS) of the mean functional image to high resolution (1 × 1 × 1 mm3) T2 structural image. This transformation was then applied to each volume in the fMRI timeseries. Finally, a bandpass filter (0.01-0.1 Hz) was applied, and images were transformed from native to MNI space with ANTS. For ADNI processing included motion correction, as well as regression of six motion parameters and mean signal from CSF and white matter. Trends were removed and a (0.01–0.08Hz) band-pass filter was applied. Images were nonlinearly registered to MNI space with coregistration to the baseline T1 image.

Volume censoring

Frames were censored based on DVARS for that frame lying outside 1.5*IQR above and below the third and first quartiles, respectively. To avoid distortion from the outlier frames, they were interpolated before bandpass filtering and then removed from the final 4D image. For ADNI after timeseries had been extracted, rows from high motion frames (> 0.5mm framewise displacement) were censored. These rows, together with one preceding and two subsequent, were removed.

Statistical modeling & inference

Model type and settings

mass univariate

Effect(s) tested

In this study, we examined how functional connectivity (FC) changes across the brain relate to age, Alzheimer's disease pathology, and cognitive function. For each factor, we computed parcelwise brain maps of effects—specifically, statistical maps showing how FC in each cortical region changes as a function of age, pathology load, or cognition. These effect maps were then compared to canonical brain gradients, which capture the large-scale organization of the cortex along functional axes (e.g., from sensory to associative regions). By correlating the spatial patterns of FC alterations with these gradients, we assessed whether changes follow systematic, cortex-wide organizational principles rather than being confined to specific networks or regions. So the effects that are actually tested are these similarities (Pearson correlation) of the brain maps.

Specify type of analysis: ☐ Whole brain ☒ ROI-based ☐ Both

Statistic type for inference

(See Eklund et al. 2016)

Specify voxel-wise or cluster-wise and report all relevant parameters for cluster-wise methods.

Correction

Describe the type of correction and how it is obtained for multiple comparisons (e.g. FWE, FDR, permutation or Monte Carlo).

Models & analysis

n/a	Involved in the study
☐	☒ Functional and/or effective connectivity
☒	☐ Graph analysis
☒	☐ Multivariate modeling or predictive analysis

Functional and/or effective connectivity

In both BioFINDER and ADNI, parcel-wise timeseries were extracted using the 1000-region Schaefer atlas, a cortical parcellation based on functional connectivity patterns and spatial contiguity. The timeseries were smoothed using a full-width at half-maximum kernel of 6 mm in BioFINDER and 4 mm in ADNI. After mean centering and scaling the raw timeseries, pairwise Pearson correlations were computed between parcels, resulting in 1000×1000 correlation matrices representing co-activation patterns. Negative correlations were set to zero. FC was quantified primarily as nodal affinity, where each connectome was column-wise thresholded to retain the 25% strongest connections, and cosine similarity was calculated between columns to yield a matrix of connectivity similarity. This matrix was then columnwise averaged, yielding one value per region, per subject.

Graph analysis

Report the dependent variable and connectivity measure, specifying weighted graph or binarized graph, subject- or group-level, and the global and/or node summaries used (e.g. clustering coefficient, efficiency, etc.).

Multivariate modeling and predictive analysis

Specify independent variables, features extraction and dimension reduction, model, training and evaluation metrics.
